# Two *Medicago truncatula* growth-promoting rhizobacteria capable of limiting in vitro growth of the *Fusarium* soil-borne pathogens modulate defense genes expression

**DOI:** 10.1007/s00425-023-04145-9

**Published:** 2023-05-12

**Authors:** Piotr Karczyński, Anna Orłowska, Ewa Kępczyńska

**Affiliations:** Institute of Biology, University of Szczecin, Wąska 13, 71-415 Szczecin, Poland

**Keywords:** Antifungal activity, Bacterial glucanase activity, Expression of genes in response to PGPR, Fabaceae, Plant growth-promoting rhizobacteria

## Abstract

**Main conclusion:**

PGPRs: *P. fluorescens* Ms9N and *S. maltophilia* Ll4 inhibit in vitro growth of three legume fungal pathogens from the genus *Fusarium*. One or both trigger up-regulation of some genes (*CHIT, GLU, PAL, MYB, WRKY*) in *M. truncatula* roots and leaves in response to soil inoculation.

**Abstract:**

*Pseudomonas fluorescens* (referred to as Ms9N; GenBank accession No. MF618323, not showing chitinase activity) and *Stenotrophomonas maltophilia* (Ll4; GenBank accession No. MF624721, showing chitinase activity), previously identified as promoting growth rhizobacteria of *Medicago truncatula*, were found, during an in vitro experiment, to exert an inhibitory effect on three soil-borne fungi: *Fusarium culmorum* Cul-3, *F. oxysporum* 857 and *F. oxysporum* f. sp*. medicaginis* strain CBS 179.29, responsible for serious diseases of most legumes including *M. truncatula*. *S. maltophilia* was more active than *P. fluorescens* in suppressing the mycelium growth of two out of three *Fusarium* strains. Both bacteria showed β-1,3-glucanase activity which was about 5 times higher in *P. fluorescens* than in *S. maltophilia.* Upon soil treatment with a bacterial suspension, both bacteria, but particularly *S. maltophilia*, brought about up-regulation of plant genes encoding chitinases (*MtCHITII, MtCHITIV, MtCHITV),* glucanases* (MtGLU)* and phenylalanine ammonia lyases* (MtPAL2*, *MtPAL4, MtPAL5).* Moreover, the bacteria up-regulate some genes from the *MYB* (*MtMYB74*, *MtMYB102*) and *WRKY* (*MtWRKY6*, *MtWRKY29*, *MtWRKY53*, *MtWRKY70*) families which encode TFs in *M. truncatula* roots and leaves playing multiple roles in plants, including a defense response. The effect depended on the bacterium species and the plant organ. This study provides novel information about effects of two *M*. *truncatula* growth-promoting rhizobacteria strains and suggests that both have a potential to be candidates for PGPR inoculant products on account of their ability to inhibit in vitro growth of *Fusarium* directly and indirectly by up-regulation of some defense priming markers such as *CHIT, GLU* and *PAL* genes in plants. This is also the first study of the expression of some *MYB* and *WRKY* genes in roots and leaves of *M. truncatula* upon soil treatment with two PGPR suspensions.

**Supplementary Information:**

The online version contains supplementary material available at 10.1007/s00425-023-04145-9.

## Introduction

To achieve higher yields necessary to feed the ever-growing human population, agriculture relies to date mainly on synthetic fertilizers and pesticides. It is projected that the global population can grow from an estimated 7.7 billion people worldwide in 2019 to about 9.7 billion in 2050 and to almost 11 billion in 2100 (United Nations 2019). Interest in high-quality and healthy agricultural products is increasing, fueled by public concerns over the use of synthetic chemicals in the environment and by the need to find alternatives to those compounds. The key to the success is a faster development of new techniques such as the low-input microbial biotechnology involving the use of beneficial microorganisms, including plant growth-promoting rhizobacteria (PGPR) which exhibit a broad spectrum of activity (Glick 2012; Barea 2015; Backer et al. 2018). There are many bacteria species that promote plant growth directly as biofertilizers and biostimulators and as biological control agents. Biocontrol involves numerous mechanisms. These include (i) secretion, by rhizobacteria, of bioactive molecules, e.g., antifungal compounds, including cell-wall degrading enzymes (chitinases and β-1-3-glucanase), (ii) competition with fungal pathogens for niche nutrients (e.g. production of siderophores), and (iii) stimulation of plant defensive capacity [e.g. via the phenylalanine ammonia lyase (PAL) activity and the production of natural compounds with antifungal properties] (van Loon et al. 1998; Glick 2012; Meena et al. 2020). It is already known that pre-treating plants with various abiotic natural and synthetic compounds, called elicitors, or with biotic agents such as PGPR can elicit induced systemic resistance (ISR) (Jakab et al. 2001; Thakur and Sokhal 2013; Król et al. 2015; Rodriguez et al. 2019). Such treatments, preceding a pathogen infection, and variously called ‘priming’, ‘conditioning’ or ‘sensitization’, reduce the severity of the disease (Conrath 2011). The physiological state induces the responsiveness of the plant’s immune system and provides non-specific protection against a wide range of stresses, both biotic and abiotic. Bacteria differ in their ability to develop ISR in plants, with some showing plant species specificity; this ability appears to depend on the specificity of the interaction between PGPR and plants (van Loon 2007; Wang et al. 2021).

Over the last decade, significant progress has been made in understanding the molecular mechanism of the PGPR-mediated disease resistance in plants (Verhagen et al. 2004; Pozo et al. 2008; van der Ent et al. 2008; Zamioudis et al. 2014; Sharma et al. 2019). ISR is phenotypically similar to the systemic acquired resistance (SAR) in that both may suppress a plant disease. However, they differ in the signaling transduction pathway; while ISR is considered as jasmonic acid (JA)/ethylene dependent, SAR is salicylic acid (SA)-dependent (van Loon 2007; Pieterse et al. 2014). ISR may be activated by certain molecules, e.g., siderophores, secreted by microorganisms referred to as elicitors (de Vleesschauwer et al. 2008; Aznar and Dellagi 2015). The induced resistance activated via ISR or SAR depends on transcription factors (TFs) from MYB and WRKY families and the expression of genes coding, *inter alia*, some enzymes associated with the immune system, such as chitinases, glucanases and PAL (Pieterse et al. 2014; Zamioudis et al. 2014; Berendsen et al. 2015; Stringlis et al. 2018; Sharma et al. 2019). Most of the information has been provided by studies on the weed *Arabidopsis*, a valuable model plant to study the priming of ISR by the PGPR *Pseudomonas simiae* (WCS417), a well-studied PGPR model (van der Ent et al. 2008, 2009; Zamioudis et al. 2013, 2014; Berendsen et al. 2015; Pieterse et al. 2021). However, a more appropriate and agronomically relevant substitute of *Arabidopsis thaliana* as a model plant for the Fabaceae, the third largest family among angiosperms and second only to the Graminae in their importance for humans (Graham and Vance 2003) has emerged in the form of the annual *Medicago truncatula*. The close phylogenetic relationship of this species to other legumes such as alfalfa, peas, lupin, faba bean, lentil and chickpea should allow a comparative analysis of genes involved in the resistance to fungal pathogens including *Fusarium* (Tivoli 2006). However, there is insufficient information on the molecular response of *M. truncatula* as a consequence of PGPR root inoculation. Although Sanchez et al. (2005) found 58 genes to be up-regulated in response to inoculation of roots of *M. truncatula* with the PGPR strain *Pseudomonas fluorescens* C7R12, there is no information to date about the expression of these genes known to participate in ISR and SAR in *M. truncatula* roots and leaves. It is not known, either, whether the plant reacts in the same way to 2 different PGPR bacteria.

We had previously identified, and registered in GenBank, 33 PGPR strains including *Pseudomonas fluorescens* (Ms9N) and *Stenotrophomonas maltophilia* (Ll4) from the rhizosphere and nodules of alfalfa, barrel medic (*Medicago truncatula*), bean and lupin. We characterized them and showed their growth-promoting effect on *Medicago truncatula* (Kisiel and Kępczyńska 2016; Kępczyńska and Karczyński 2020). To our knowledge, effects of these bacteria on in vitro growth of soil-borne *Fusarium* fungi have not been investigated yet. There are no studies either on the effects of the bacteria in the expression of defense marker genes (*CHIT*, *GLU*, *PAL*) in *M. truncatula* roots and leaves. Moreover, the biological control efficiency depends on three variables: PGPR, the pathogen and the host plant genotype. It has been recently stated that biological control should be based on the use of a bacterial consortium rather than on a single bacterial strain. Hence, identification of any new potential rhizobacterial isolate and understanding its direct and indirect biocontrol activity is a contribution to sustainable agriculture (Meena et al. 2020). Here, we broaden the understanding of the molecular roots-to-leaves response in *M. truncatula* as a consequence of soil treatment with suspensions of *Pseudomonas fluorescens* (Ms9N) and *Stenotrophomonas maltophilia* (Ll4); both PGPR bacteria are capable of producing siderophores, but only Ll4 have chitinase activity (Kępczyńska and Karczyński 2020)**.** Thus, the present study was aimed at (i) finding out whether the two *M. truncatula* PGPB identified earlier, namely *Pseudomonas fluorescens* (Ms9N) and *Stenotrophomonas maltophilia* (Ll4) are capable of directly controlling the mycelial growth of three legume soil-borne fungi: *Fusarium culmorum* Cul-3, *F. oxysporum* 857 and *F. oxysporum* f. sp. *medicaginis* CBS179.29 under in vitro conditions; and (ii) ascertaining whether the bacteria can modulate, in *M. truncatula* roots and leaves, expression of genes known to be associated with the defense response, such as *CHIT* (*MtCHITI*, *MtCHITII*, *MtCHITIII*, *MtCHITIV, MtCHITV*), *MtGLU* and *PAL* (*MtPAL1*, *MtPAL2*, *MtPAL4*, *MtPAL5*) encoding chitinases, glucanase and phenylalanine ammonia lyases, respectively. In addition, we tried to check whether the genes from the *MYB* and *WRKY*  families (*MtMYB74, MtMYB102*, *MtWRKY6, MtWRKY29*, *MtWRKY53* and *MtWRKY70*) we selected respond, in roots and leaves, to soil inoculation.

## Material and methods

### Fungal and bacterial species, preparation of pathogen inocula, and *Medicago truncatula* plant growth

*Fusarium culmorum* strain Cul-3 and *F. oxysporum* 857 were obtained from the collection of the Institute of Plant Genetics (Polish Academy of Sciences, Poznań, Poland), while *F. oxysporum* f. sp. *medicaginis* strain CBS179.29 was purchased from the Centraalbureau voor Schimmelcultures (Utrecht, The Netherlands). The *Fusarium* strains were grown in Petri dishes in the dark on 2% potato dextrose agar (PDA; Difco Laboratories) at 28 ˚C for 14 days. Fungal inocula consisted of agar discs (0.5 cm diameter) punched out with a sterilized corkborer from the edges of the growing colonies.

### Cultivation of bacterial strains and inoculum preparation

The PGPR bacterial strains used in the study represented two families: the Pseudomonadaceae [*Pseudomonas fluorescens* (Ms9N); GenBank accession No. MF618323) isolated earlier from nodules of *Medicago sativa*, and the Xantomonadaceae [*Stenotrophomonas maltophilia* (Ll4); GenBank accession No. MF624721] isolated from the rhizosphere of *Lupinus luteus* (Kępczyńska and Karczyński 2020). The bacteria were placed in Eppendorf tubes in a mixture of the bacterial medium (LB, Scharlau, Scharlab, S.L. Spain) and 25% glycerol solution, and were stored at -80ºC. The inoculum of bacteria from the rhizosphere and nodules was prepared by growing bacterial cells in 20 ml of liquid TSB and 2xYT medium (both from OXOID Ltd., Basingstoke, Hampshire, UK) and incubating them in a shaker incubator (200 rpm) at 28 ºC. The density of each culture was measured in a Shimadzu UV–Vis 1800 spectrophotometer at 600 nm. The medium was subsequently separated from the culture by centrifugation (8000* g*/10 min/4 ºC). The cells were suspended in 20 ml of sterile 10 mM MgSO_4_. Following centrifugation, the supernatant was discarded, and the washing procedure was repeated twice. The cell suspension was diluted 20 times by adding sterile 10 mM MgSO_4_, 10 ml portions of the dilution being used to inoculate the plants, as described below.

### Plant growth and treatment conditions

The *Medicago truncatula* Gaertn. Jemalong ecotype J5 plants (seeds provided by French National Institute for Agricultural Research—INRA) were cultivated in pots with a soil mixture consisting of sand and perlite (1:1, w/w) in a growth room under controlled conditions with light–dark and temperature cycles of 16 h light at 24 ºC; 8 h dark at 20 ºC. The light density was 150 μmol m^−2^ s^−1^ (Green Power LED modules, Philips). The seedlings were obtained and cultivated as described in detail in an earlier paper (Kępczyńska and Karczyński 2020). For ISR induction, a suspension of *Pseudomonas fluorescens* (Ms9N) and *Stenotrophomonas maltophilia* (Ll4) in 10 mM MgSO_4_ was applied (10 ml) to 4 week-old plants, 1 cm away from the stem, with a pipette. As the control, 10 mM MgSO_4_, was applied in a similar manner. 24 h and 72 h after soil treatment, the plants were harvested and the soil adhering to the roots was removed by gently washing them in sterile water. The roots and leaves to be used in the gene expression analysis were separated from the shoots and were immediately frozen in liquid nitrogen and stored at –80 ºC. Molecular analyses were also performed on roots and leaves of 4-week-old seedlings not inoculated and not treated with 10 mM MgSO_4_ solution alone (before treatments).

### In vitro antifungal activity of *P. fluorescens* and *S. maltophilia*

Both bacteria were tested for their potential to act as biocontrol agents against *M. truncatula* soil-borne pathogens: *Fusarium culmorum* Cul-3*, F. oxysporum* 857 and *F. oxysporum* f. sp. *medicaginis* CBS 179.29. The *Fusarium* strains were grown on PDA at 28 ºC in the dark. 5-mm discs from a 2 week-old sporulating mycelia were placed in the center of the PDA medium dish (10 cm in diameter). Subsequently, 30 µl portions of the bacterial suspension tested were spread with a loop on both sides of the dish (1 cm from the edge). This experiment involved a 2 day-old liquid bacterial suspension (LB medium, 10^6^ CFU/ml) obtained from a bacterial pre-culture in our laboratory’s microbial bank. The control consisted of bacteria-free mycelial cultures. All the variants were repeated 3 times. At specified time points, the cultures were photographed, and the mycelial area was calculated using ImageJ software (National Institutes of Health and the Laboratory for Optical and Computational Instrumentation, University of Wisconsin).

### Determination of bacterial β-1,3-glucanase activity

β-1,3-Glucanase (GLU) activity was measured according to the methodology of Lim et al. (1991) with some modifications. To activate *Pseudomonas fluorescens* (Ms9N) and *Stenotrophomonas maltophilia* (Ll4) β-1,3-glucanase activity, they were grown on the liquid M9 medium supplemented with 0.02% laminarin (from *Laminaria digitata*, Sigma) at 28 °C for 72 h on a rotary shaker (140 rpm). Subsequently, the bacteria were centrifuged (8000* g*, 6 min, 4 °C), the supernatant was decanted, and the bacteria were suspended in 0.1 M phosphate buffer (pH 5.5). To concentrate the enzyme, the bacteria were centrifuged again (25,000* g*, 20 min, 4 °C) and the supernatant obtained was poured into new test tubes.

To determine the enzyme activity, the release of reducing sugars was measured with the Nelson (1944). The reaction mixture consisted of 400 µl of enzyme extract and 250 µl of 0.2% laminarin. Additionally, to determine the glucose content in extracts and laminarin thermal decomposition, tests were made without laminarin (with water) and without the extract (with phosphate buffer). Samples were incubated at 40 °C for 2 h. The enzyme activity unit (U) was 1 µmol of glucose produced by 1 mg of enzyme for 1 h.

### Phylogenetic identification

The amino acid sequences of MYB74, MYB102 and 4 WRKY proteins from the *A. thaliana* database (https://www.arabidopsis.org/) were used as queries to perform a BLASTp search against the NCBI database (https://www.ncbi.nlm.nih.gov/). Specific domain locations were confirmed by reference to the Pfam (https://pfam.xfam.org/) and InterProScan (https://www.ebi.ac.uk/interpro/) databases. ClustalW tools of the Geneious 6.1 software (https://www.geneious.com/, Kearse et al. 2012) were used for the alignment of protein sequences. The phylogenetic trees based on full-length protein sequences were constructed using the Neighbor-Joining and Maximum Likelihood methods with 1000 bootstrap replicates.

### Gene expression analysis

Total RNA was extracted from the root and leaf tissue collected from 4 week-old plants at 3 time points: before (0 h) and after inoculation (24 h and 72 h), with TRIzol Reagent (GenoPlast) and was purified by Direct-zol™ RNA-MiniPrep kit (Zymo Research). The first-strand cDNA was synthetized from 500 ng of RNA samples using the NG dART RT kit (EURx). Quantitative real-time PCR (qPCR) was performed using the Step-One™ Real-Time PCR System (LifeTechnologies) with the 5 × HOT FIREPol^®^ EvaGreen^®^ qPCR Mix Plus (ROX) (Solis BioDyne) following the manufacturer’s instruction, as described earlier by Kępczyńska and Karczyński (2020). The expression levels were normalized to *ACTIN2* using the 2^−∆∆CT^ method (Livak and Schmittgen 2001). The qPCR experiments were run with 3 biological and technical replicates for each sample. Primers (Table S1) were designed using the PrimerExpress^®^ Software v3.0 (LifeTechnologies) and their gene specificity was checked with Primer-BLAST (NCBI).

### Statistical analysis

All the experiments were run with at least 3 biological replicates; the results are expressed as the mean ± standard deviation (SD). Statistical analyses were performed using one-way or two-way ANOVA, followed by Tukey’s HSD post-hoc test.

## Results

### In vitro growth inhibition of *Fusarium* species in the presence of *Pseudomonas fluorescens* Ms9N and *Stenotrophomonas maltophilia* Ll4

**Fig. 1:**
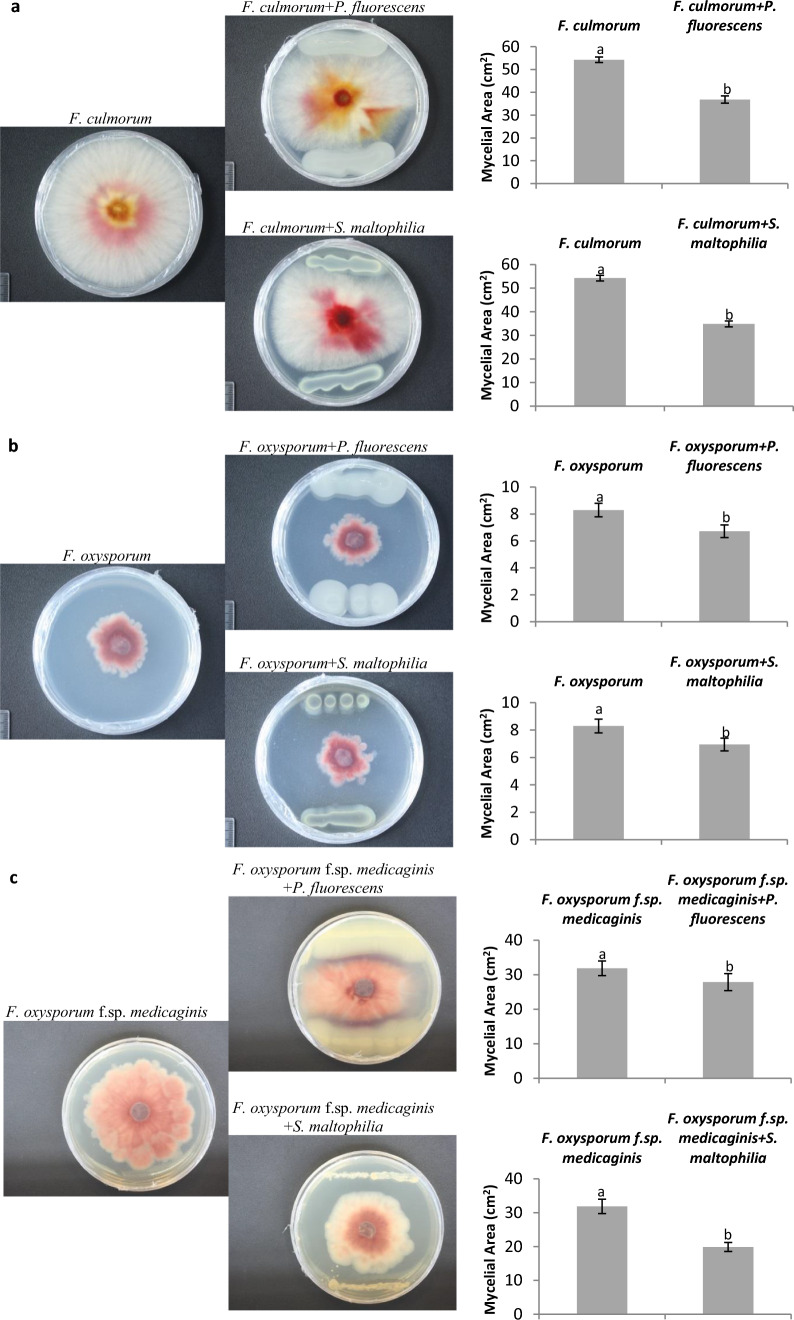
Effects of *P. fluorescens* Ms9N and *S. maltophilia* L14 on in vitro growth of *F. culmorum* after 4 days (**a**), *F. oxysporum* after 5 days (**b**) and *F. oxysporum* f. sp. *medicaginis* after 9 days (**c**). Data are means ± SD (*n* = 3) and the results were replicated in at least 3 independent experiments. Letters denote significance of differences, as determined by one-way ANOVA (*P* < 0.05), followed by Tukey’s HSD post-hoc test

The two PGPR species selected for the study, previously identified as promoting growth of *Medicago truncatula* (Kępczyńska and Karczyński 2020), inhibited growth of *Fusarium culmorum* mycelium (Fig. 1a). After 4 days, *P. fluorescens* produced a distinct area of pathogen mycelium inhibition (32%). During the same time, the other bacteria studied, *S. maltophilia*, reduced growth of *F. culmorum* even more as the mycelial area was by 36% smaller than the control mycelium. In addition, the two bacteria inhibited growth of another *Medicago* fungal pathogen, *F. oxysporum* 857 (Fig. 1b). Although growth of that fungus was much weaker under identical conditions, its (control) surface was more than 8 times smaller compared to the surface of *F. culmorum* (Fig. 1a). The inhibitory effect of the two PGPRs on mycelial growth was observed as soon as on day 5 (Fig. 1b): *P. fluorescens* and *S. maltophilia* produced 19% and 16% inhibition, respectively. The bacteria, but especially *S. maltophilia*, exerted an inhibitory effect on growth of *F. oxysporum* f. sp. *medicaginis*, the fungus growth being inhibited by almost 40% (Fig. 1c). These results indicate that the bacteria isolated from *Medicago sativa* nodules (*P. fluorescens* Ms9N) and from the rhizosphere of *Lupinus luteus* (*S. maltophilia* Ll4) do possess some potential for a direct biological control of soil-borne Fabaceae fungal pathogens of the genus *Fusarium*.

### The β-1,3-glucanase activity of selected bacteria

**Fig. 2:**
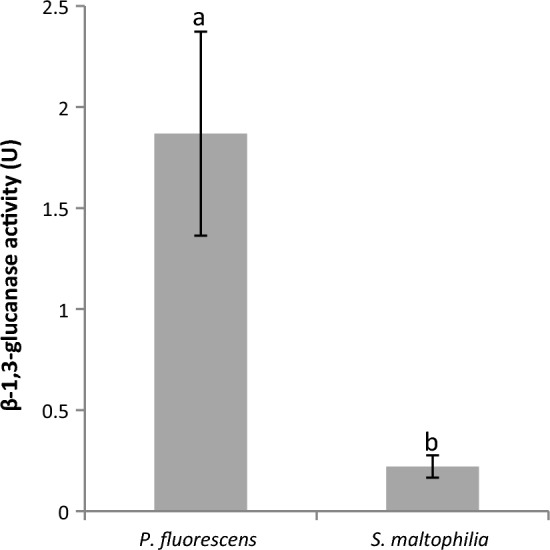
β-1,3-glucanase activity in *P. fluorescens* Ms9N and *S. maltophilia* Ll4 after 3 days in the peptone medium, supplemented with 0.02% laminarin. Data are means ± SD (*n* = 3) and the results were replicated in at least 3 independent experiments. Letters denote significance of differences, as determined by one-way ANOVA (*P* < 0.05), followed by Tukey’s HSD post-hoc test

Since *P. fluorescens* (a siderophore producer) and *S. maltophilia* (a producer of siderophores and chitinases) inhibited the in vitro mycelial growth of *Fusarium* species in this study, we checked if the bacteria were also have a β-1,3-glucanase activity, an enzyme responsible for hydrolysis of glucan which, along with chitin, is the main component of fungal cell walls. The two bacteria proved have a β-1,3-glucanase activity (Fig. 2); the enzyme’s activity in *P. fluorescens* was about 5 times higher than in *S. maltophilia*.

The data on the direct control, presented above, show that the inhibitory effect of bacteria on the in vitro growth of *Fusarium* may be related to the ability of these bacteria to secrete enzymes that hydrolyze the main components of the fungal cell walls.

### Expression of chitinase, β-1,3-glucanase and PAL encoding genes in *M. truncatula* roots and leaves and its modification by soil treatment

**Fig. 3:**
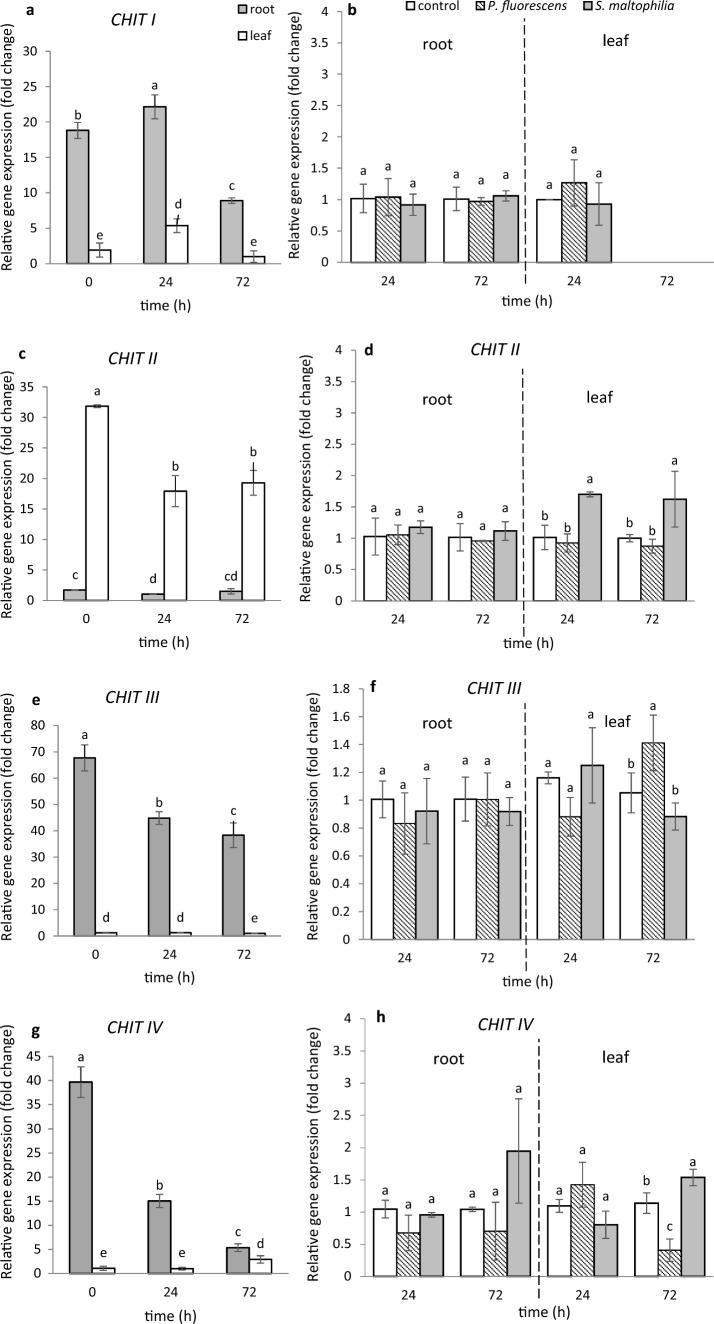

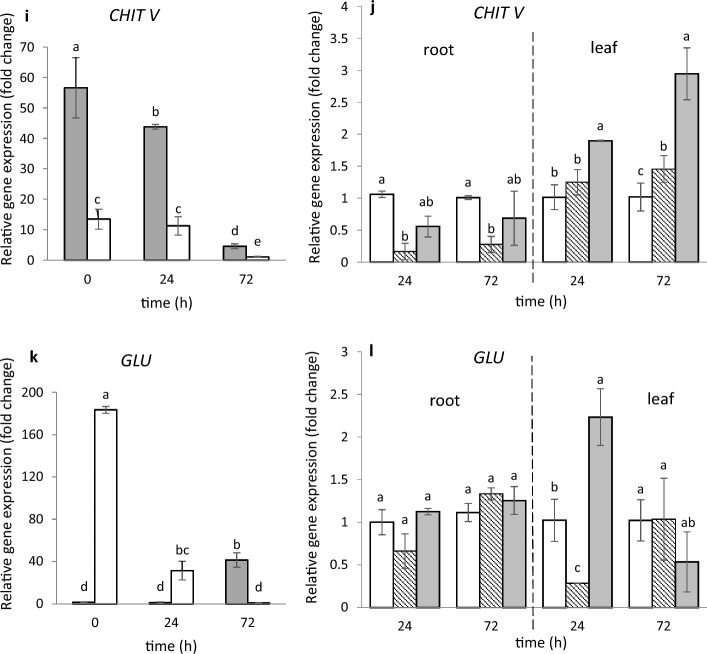
Expression of *CHIT I*, *CHIT II*, *CHIT III*, *CHIT IV*, *CHIT V* and *GLU* genes in *M. truncatula* roots and leaves before, 24 h and 72 h after treating the soil with MgSO_4_ solution (**a**, **c**, **e**, **g**, **i**, **k**) and after treatment with *P. fluorescens* Ms9N and *S. maltophilia* L14 suspensions (**b**, **d**, **f**, **h**, **j**, **l**). Expression in roots and leaves after treating the soil with MgSO_4_ (left side) measured relative to the lowest observed expression taken as 1; expression in roots and leaves after treating the soil with bacterial suspensions (right side) measured relative to the control samples (roots and leaves after soil treatment with MgSO_4_ only) taken as 1. At least 3 biological replicates were performed for all experiments and are shown as the mean ± SD. Statistical analyses were performed using two-way ANOVA, followed by Tukey’s HSD post-hoc test

To investigate whether the bacteria tested, capable of directly controlling the fungi in vitro*,* can be regarded as protecting the plant from a possible attack of fungal pathogens, we checked if they would change expression of genes known as markers of defense pathways, in *M. truncatula* roots and leaves located away from the place of colonization. From the chitinase-encoding genes, identified previously in *M. truncatula* (Salzer et al. 2000), 5 representing 5 classes (I, II, III, IV, V) – were selected [*Mtchitinase I (MtCHITI), Mtchitinase II (MtCHITII), Mtchitinase III (MtCHITIII), Mtchitinase IV (MtCHITIV)* and *Mtchitinase V (MtCHITV)*]*.* Constitutive expression of these genes (time 0) took place in 4-week-old roots and leaves* (*Fig. 3a, c, e, g, i). Among the 5 genes tested at time 0 as many as 4 showed a much higher level of expression in roots compared to the leaves; this was particularly distinct with respect to *MtCHITIII* and *MtCHITIV,* the increase being about 56- and 40-fold, respectively (Fig. 3a, e, g, i). However, in the case of *MtCHITII,* the constitutive expression in leaves was nearly 19 times higher than that in roots (Fig. 3c). In control seedlings, the expression of all tested genes in roots and leaves changed during 72 h. Soil inoculation with *S. maltophilia* Ll4 suspension did not change the expression of the 5 tested *chitinases* genes in the roots within 72 h (Fig. 3b, d, f, h, j). Also, no changes in the expression of 4 out of 5 genes, caused by *P. fluorescens* Ms9N suspension were observed in roots (Fig. 3b, d, f, h); even down-regulation of the *MtCHITV* has been noted (Fig. 3j). However in leaves 72 h upon soil inoculation by *S. maltophilia* up-regulation of *MtCHITII*, *MtCHITIV* and *MtCHITV* was noted (Fig. 3d, h, j). Also, *P. fluorescens* Ms9N was found to increase the expression of two genes: *MtCHITIII* and *MtCHITV* in leaves after 72 h after inoculation (Fig. 3f, j). Thus, it was only in leaves 72 h after inoculation that one gene, *MtchitinaseV* was up-regulated by both bacteria; a stronger up-regulation was effected by *S. maltophilia* Ll4 which, unlike *P. fluorescens* Ms9N, has the ability to secrete chitinase.

The same experiment explored expression of the gene coding β-1,3-glucanase (*GLU)* responsible for the hydrolysis of β-1,3-glucan, a fungal cell-wall component. The constitutive expression (time 0) in 4-week-old *M.truncatula* plants of *MtGLU* was 108 times higher in leaves than in the roots and changed during 72 h after soil treatment with salt solution (controls) (Fig. 3k). Soil treatment with the suspensions of both bacteria had no effect on the expression of the *MtGLU* gene in roots. In leaves *S. maltophilia* caused up-regulation of this gene; twofold increase its expression after 24 h is observed (Fig. 3l).

**Fig. 4:**
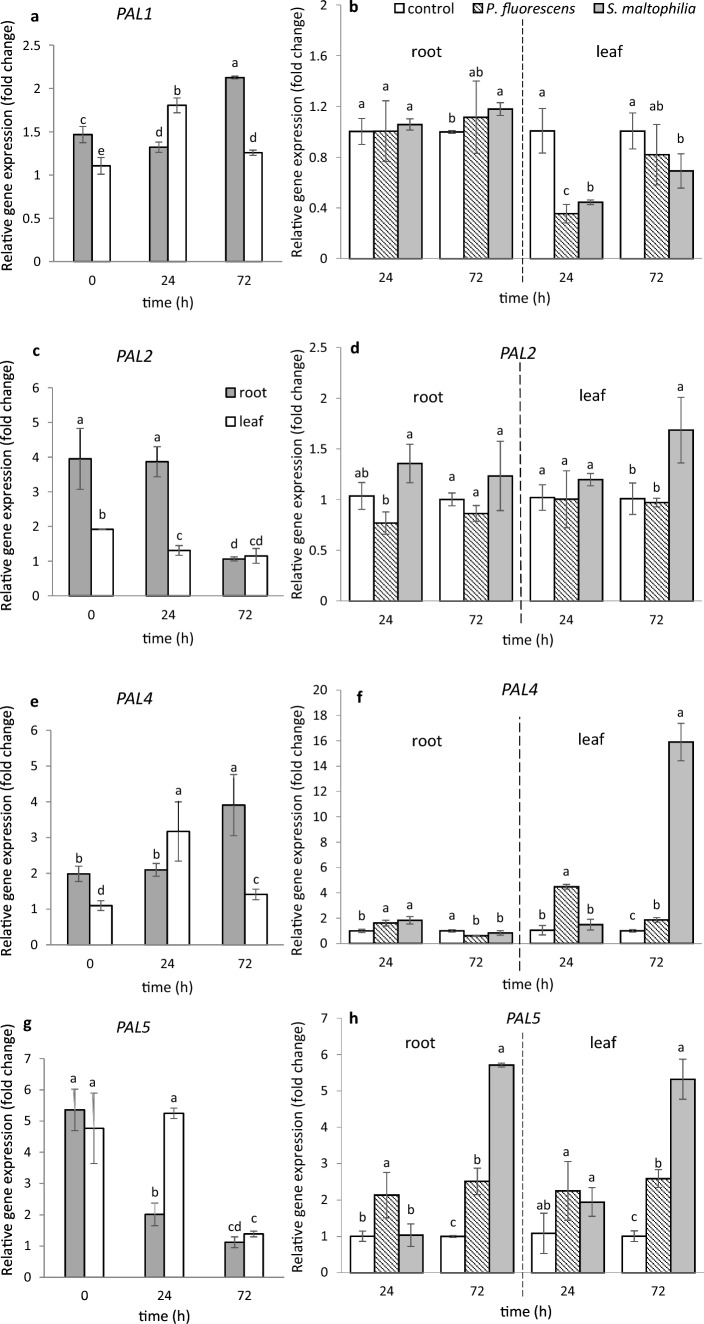
Expression of *PAL1*, *PAL2*, *PAL3*, *PAL4* and *PAL5* genes in *M. truncatula* roots and leaves before, 24 h and 72 h after treating the soil with MgSO_4_ solution (**a**, **c**, **e**, **g**) and after treatment with *P. fluorescens* Ms9N and *S. maltophilia* L14 suspensions (**b**, **d**, **f**, **h**). Expression in roots and leaves after treating the soil with MgSO_4_ (left side) measured relative to the lowest observed expression taken as 1; expression in roots and leaves after treating the soil with bacterial suspensions (right side) measured relative to the control samples (roots and leaves after soil treatment with MgSO_4_ only) taken as 1. At least 3 biological replicates were performed for all experiments and are shown as the mean ± SD. Statistical analyses were performed using two-way ANOVA, followed by Tukey’s HSD post-hoc test

Since PAL is known to be a defense-related enzyme, we also checked whether the genes encoding this enzyme are expressed in roots and leaves of *M. truncatula* in response to soil inoculation by both bacteria*.* For the analysis, we selected 4 out of the 6 *PAL* genes identified in the genome of *M. truncatula* by Ren et al. (2019) (Fig. 4). All the 4 genes were expressed in roots and leaves of 4-week-old seedlings (time 0), with the expression of the *MtPAL1, MtPAL2* and *MtPAL4* genes being higher in the roots than in the leaves (Fig. 4a, c, e); expression of *MtPAL5* in these organs was on the same level (Fig. 4g). Expression profile of all genes changed during 72 h upon soil treatment with salt solution (control). The two bacteria differed in their effect on the expression profile of the 4 *PAL* genes tested (Fig. 4b, d, f, h). Roots showed the largest changes in the *MtPAL5* gene expression; 72 h after inoculation, a clear up-regulation took place under the influence of both bacteria (Fig. 4h). In leaves also both bacteria after the same time caused up-regulation of this gene. Up-regulation by both bacteria was also observed in case of *MtPAL4* (Fig. 4f). After 72 h a particularly high increase, about 16-fold compared to the expression of *MtPAL4* in leaves of plants growing in non-inoculated soil, was found to have been produced by *S. maltophilia* Ll4. This bacteria also caused up-regulation of *MtPAL2* (Fig. 4d). These results clearly show that the *P. fluorescens* Ms9N and *S. maltophilia* Ll4 strains tested produced different effects on the expression profile of the *PAL* genes in roots and leaves after soil inoculation.

### Phylogenetic analysis and domain distribution of MYB74, MYB102 and 4 WRKY proteins in *M. truncatula*

Many studies indicate that the MYB and WRKY TFs play a very important part at different stages of plant development, including their essential roles in regulations of gene expression to cope with abiotic and biotic environmental factors (Li et al. 2004, 2019a; Dubos et al. 2010; Wang and Li 2017; Backer et al. 2019). Since our research involved both abiotic and biotic factors, it was interesting to check whether they would modulate expression of the genes we selected from the two families in *M. truncatula* roots and leaves. Prior to the expression analysis of the TFs coding genes such as MYB and WRKY, it was necessary to find *A. thaliana* homologues in *M. truncatula*. *At*MYB72 is known for its role in ISR (van der Ent et al. 2008), but we were unable to identify *MYB72* in *M. truncatula* genome, so for further analysis we selected genes that are also known to be involved in the response to abiotic and biotic environmental factors, i.e. *MYB74* and *MYB102* (Denecamp and Smeekens 2003; de Vos et al. 2006; Ortiz-Garcia et al. 2022). Although MYB102 has already been identified in *M. truncatula* by Wang and Li (2017), we selected both TFs for the analysis because of the amino acid sequence similarity of the *Arabidopsis* MYB74 protein, a paralog of MYB102.

The search for MYB74, MYB102 and 4 WRKY homologous proteins was based on the amino acid sequence similarity and the organization of protein domains. The BLASTp analysis of the *Mt*MYB74 amino acid sequence showed it to be highly homologous with MYB74 (94%) and MYB102 (92%) of *A. thaliana,* but the homology with *Mt*MYB102 was as low as 54%. Moreover, MtMYB102 showed a lower similarity with the *Arabidopsis* MYB proteins: 57% with *At*MYB102 and 77% with *At*MYB74. *Mt*MYB74 and *Mt*MYB102 were more similar to their *A. thaliana* homologs than to each other. Both *M. truncatula* MYB74 and MYB102 formed 1 clade with other MYB proteins of the family Fabaceae (Fig. S1a). All the MYB74 and MYB102 proteins of *A. thaliana* and *M. truncatula* contain two Myb-like DNA-binding domains located on N-tails (Fig. S1b).

**Fig. 5:**
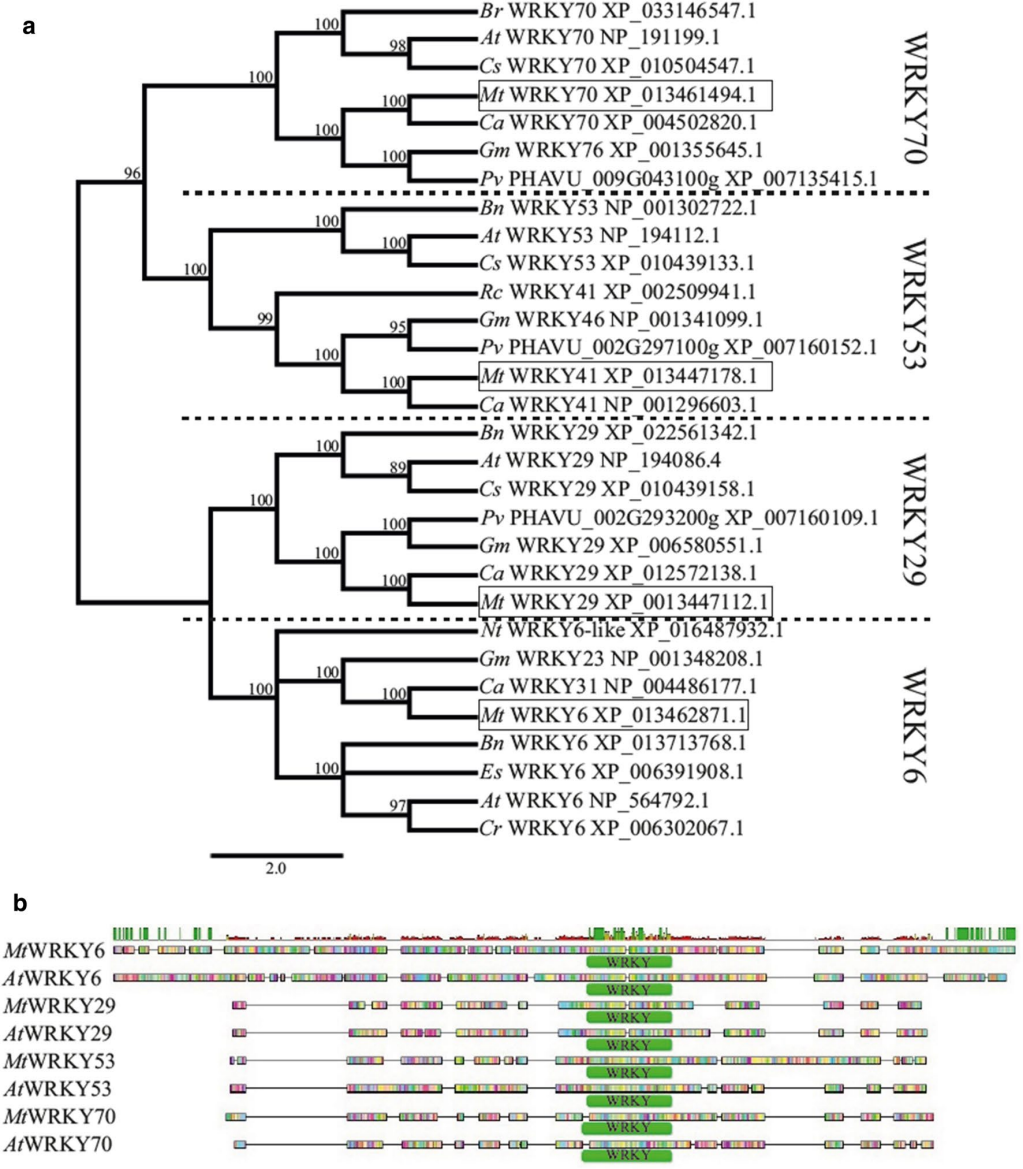
Phylogenetic trees and domain organization based on deduced amino acid sequences of *Medicago truncatula* (*Mt*) and *Arabidopsis thaliana* (*At*) WRKY6, WRKY29, WRKY53 and WRKY70 (**a**, **b**). *Bn* *Brassica napus*, Br *Brassica rapa*, Ca *Cicer arietinum*, Cr *Capsella rubella*, Cs *Camelina sativa*, Es, *Eutrema salsugineum*, Gm *Glycine max*, Nt *Nicotiana tabacum*, Pv *Phaseolus vulgaris*, Rc *Ricinus communis*

The 4 WRKY proteins (WRKY6, WRKY29, WRKY53 and WRKY70) separated into 4 distinct clades (Fig. 5a). The amino acid analysis showed all the orthologous proteins found in *A. thaliana* and the family Fabaceae to belong to the same clades. The domain organization analysis showed all the WRKY proteins of *A. thaliana* and *M. truncatula* to possess a characteristic WRKY DNA-binding domain (Fig. 5b).

### Expression of *MtMYB74*, *MtMYB102*,* MtWRKY6*, *MtWRKY29*, *MtWRKY53* and *MtWRKY70 *genes in *M. truncatula* roots and leaves and its modification upon soil treatment.

**Fig. 6:**
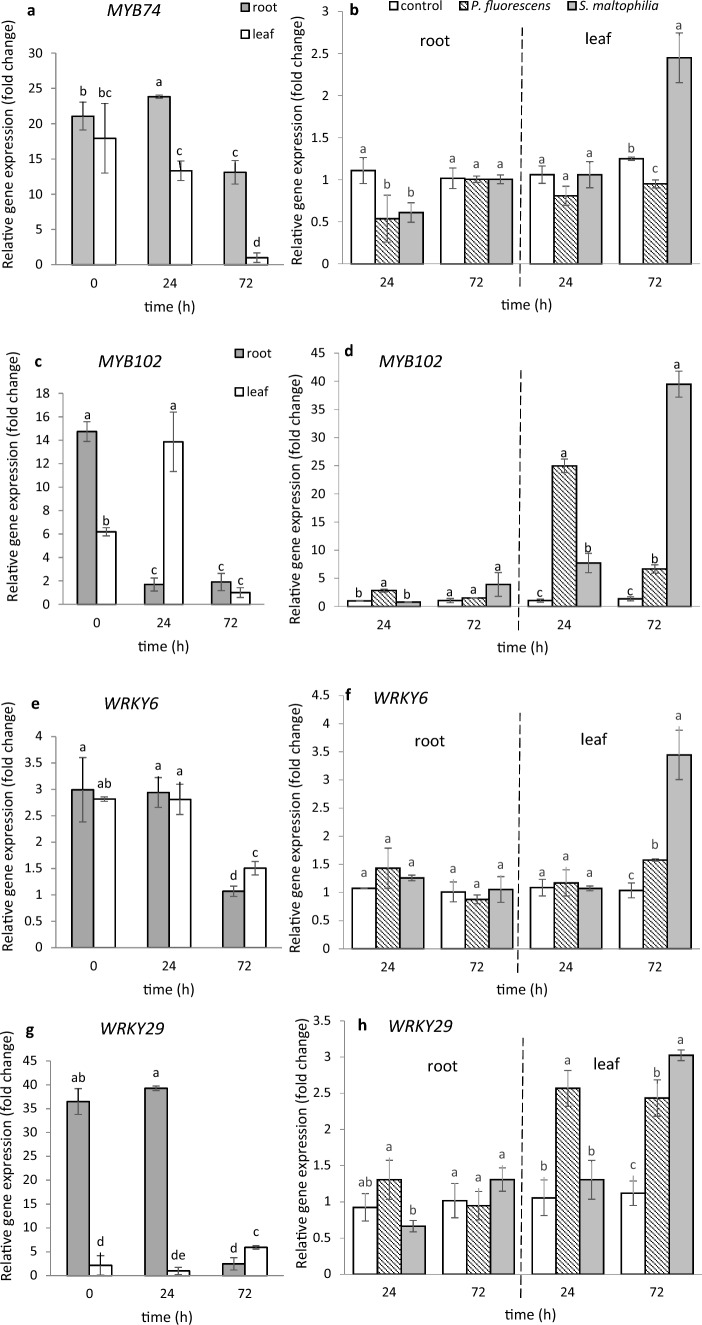

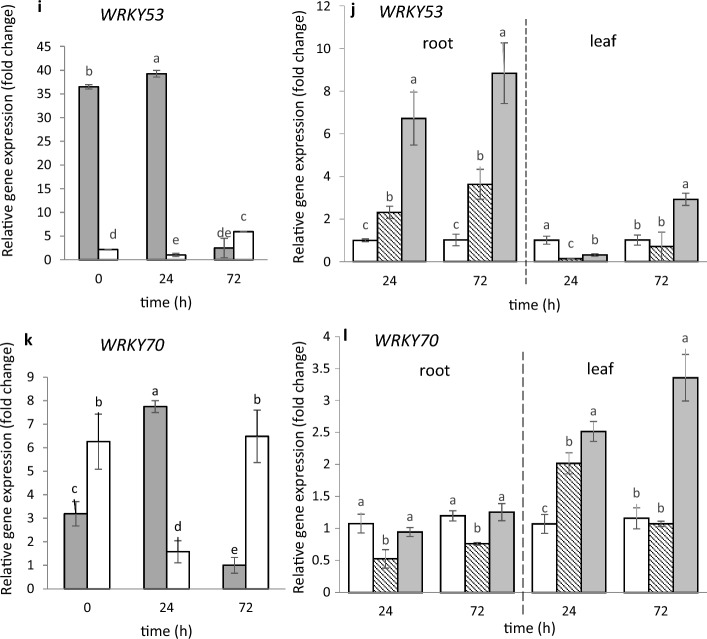
Expression of selected *MYB74*, *MYB102*, *WRKY6*, *WRKY29*, *WRKY53* and *WRKY70* genes in *M. truncatula* roots and leaves before, 24 h and 72 h after soil treatment with MgSO_4_ solution (**a**, **c**, **e**, **g**, **i**, **k**) and after treatment with *P. fluorescens* Ms9N and *S. maltophilia* L14 suspensions (**b**, **d**, **f**, **h**, **j**, **l**). Expression in roots and leaves after treating the soil with MgSO_4_ (left side) measured relative to the lowest observed expression taken as 1; expression in roots and leaves after treating the soil with bacterial suspensions (right side) measured relative to the control samples (roots and leaves after soil treatment with MgSO_4_ only) taken as 1. At least 3 biological replicates were performed for all the experiments and are shown as the mean ± SD. Statistical analyses were performed using two-way ANOVA, followed by Tukey’s HSD post-hoc test

As shown in Fig. 6a and c, constitutive expression of *MtMYB74* and *MtMYB102* in roots and leaves of untreated 4-week-old seedlings (time 0) does take place. The level of *MtMYB74* expression was similar in both organs, while the expression of *MtMYB102* in roots was more than 2 times higher than that in the leaves; expression profile of both genes changed upon soil treatment with salt solutions (control). In roots, only *P. fluorescens* Ms9N after 24 h caused up-regulation of *MtMYB102* (Fig. 6d). In turn in leaves, after 72 h, both bacteria brought about up-regulation of *MtMYB102*, a particularly strong effect being caused by *S. maltophilia* (Fig. 6d).

We conducted a similar study on expression of the *WRKY* genes coding WRKY proteins, which comprise a large family of plant TFs. Based on information regarding the *Arabidopsis* response to abiotic and biotic factors (Euglem and Somssich 2007; Jaśkiewicz et al. 2011; Wiesel et al. 2014; Nie et al. 2017), we selected 4 *WRKY* genes (*MtWRKY6, MtWRKY29, MtWRKY53, MtWRKY70)*. As shown in Fig. 6e, g, i, k, all the 4 genes were expressed in roots and leaves of 4-week-old seedlings at the start of the experiment (time 0), but the levels of the expression differed. While the expression of *MtWRKY6* in roots was at the same level as that of *MtWRKY6, MtWRKY29,* and *MtWRKY53* in leaves, the expression of *MtWRKY29* and *MtWRKY53* was higher in roots than in leaves by a factor of 18, the expression of *MtWRKY70* in roots being half that in leaves. Expression profile of all genes changed during 72 h upon soil treatment with salt solution (control). In the roots, both bacteria cause up-regulation of one of the 4 studied genes, i.e., *MtWRKY53* (Fig. 6j). Especially *S. maltophilia* Ll4, significantly stimulated expression of the gene in roots both after 24 h and 72 h after soil inoculation. In turn 72 h after inoculation, the leaves, distant from the contact site with bacteria, showed *S. maltophilia* Ll4 to have up-regulated all the 4 *WRKY* genes tested (Fig. 6f, h, j, l) and *P. fluorescens* Ms9N having up-regulated two genes, i.e., *MtWRKY6* and *MtWRKY29* (Fig. 6f, h).

## Discussion

Emerging strategies for fungal plant disease management include a biological control by application of antagonistic microorganisms, such as the PGPR, and involving their direct or indirect action. Although much progress in understanding the mechanisms of the action has been achieved during more than the past two decades, including the molecular level of biocontrol of fungal soil pathogens by PGPR (Rodriguez et al. 2019; Pieterse et al. 2021), knowledge on their role in this complex process remains very incomplete. Moreover, most of the information has come from a model of interactions between a microbe (*Pseudomonas*) and a plant (the weed *Arabidopsis thaliana*). However, such mechanisms have not been explored in economically important crop species such as the Fabaceae for which *Medicago truncatula* is a molecular model. In this paper, we described two PGPR strains promoting growth of *M. truncatula* roots and shoots (Kępczyńska and Karczyński 2020), which can also be considered as candidates for biocontrol agents against *Fusarium* species due to their ability to directly inhibit growth of the fungi. They are also capable of producing such effect indirectly by triggering activation of the defense marker genes expression, in *M. truncatula* roots and leaves.

### Direct in vitro control of three *Fusarium *species by *P. fluorescens *Ms9N and *S. maltophilia* Ll4

*P. fluorescens* Ms9N and *S. maltophilia* Ll4, i.e., 2 out of the 13 bacteria isolated from organic farms growing *M. truncatula* (Kępczyńska and Karczyński 2020) have a potential to act as biocontrol agents in reducing development of serious soil-borne fungal pathogens of the Fabaceae, including *Medicago* spp.: *F. culmorum* causing the spring black stem and root rot, *F. oxysporum* 857 and *F. oxysporum* f. sp. *medicaginis* CBS 179.29 responsible for vascular wilt. Different antifungal activities of the bacteria studied are probably related to, *inter alia*, their different ability to secrete fungal cell wall-hydrolyzing enzymes, i.e., chitinases and glucanases. Although both bacteria secrete glucanases (Fig. 2), only *S. maltophilia* Ll4 is capable of secreting chitinases (Kępczyńska and Karczyński 2020), hence probably a higher antifungal activity (suppressing the development of 2 out of the 3 *Fusarium* strains tested in vitro)*.* Kamil et al. (2007) demonstrated earlier that, under in vitro conditions, a chitinolytic isolate of *S. maltophilia* MS2 was able to inhibit mycelial growth of *Phytium* sp., another soil-borne fungal pathogen responsible for the seedling damping-off disease. Similarly, another strain of the same bacterial species, *S. maltophilia* W8 used in a dual mixture with *P. fluorescens* F113, was effective in suppressing the development of *Phytium* spp. which causes sugar beet damping-off (Jetiyanon and Kloepper 2002). The fungicidal activity of *S. maltophilia* W8 was due to extracellular proteolytic enzymes. A mixture of two strains of *Burkholderia* spp. (RHT8 and RTH12) secreted siderophores, chitinase and β-1,3-glucanase, and were also effective in reducing *F. oxysporum,* a pathogen responsible for fenugreek wilting (Kumar et al. 2017). Our results concerning the growth-inhibiting effect on the *Fusarium* species mycelium suggest that both *P. fluorescens* Ms9N and *S. maltophilia* Ll4 can be considered as good candidates for a dual usage in the control of soil-borne *Fusarium* species infecting the plant root system. The ability to produce siderophores by the two bacteria tested as well as the chitinolytic activity of *S. maltophilia* Ll4 (Kępczyńska and Karczyński 2020) and the ability of the two bacteria to secrete β-1,3-glucanase shown in in this study may be useful in direct reduction of soil fungal pathogens. Bacterial siderophores can directly deprive the pathogen of iron because the fungal siderophores have a lower iron sequestration ability (Kloepper et al. 1980; Aznar and Dellagi 2015). In addition, compounds such as pseudobactin, pyochelin and pyoverdine from *Pseudomonas* can elicit induction of immune reactions (Buysens et al. 1996; van Loon and Bakker 2007; de Vleesschauwer et al. 2008; van Loon et al. 2008; Aznar and Dellagi 2015; Berendsen et al. 2015).

### Different expression of genes encoding chitinase, β-1,3-glucanase and PAL in *M. truncatula* roots and leaves upon soil treatment

Since the bacteria tested are capable of directly controlling serious soil-borne fungal pathogens of *Medicago* sp., it was interesting to see if these bacteria are able to modulate the expression of some defense pathway marker genes encoding chitinases (categorized into pathogenesis-related proteins PR-3, − 4, − 8, − 11 families), β-1,3-glucanases from PR-2 (Vaghela et al. 2022) as well as phenylalanine ammonia lyase (PAL) responsible for activation of the phenylpropanoid pathway. In this study, expression of all the 5 selected chitinase genes (*MtCHITI*, *MtCHITII, MtCHITIII, MtCHITIV, MtCHITV*) took place in roots and leaves of 4-week-old *M. truncatula* seedlings, with a high level of expression in roots compared to leaves, except for *MtCHITII*; there, a high level of expression in leaves, compared to roots, was observed (Fig. 3). The biotic agent used in this study did not change the expression, in roots, of 4 out of the 5 genes tested; it was only under the influence of *P. fluorescens* that down-regulation of *MtCHITV* took place. However, three genes (*MtCHITII, MtCHITIV, MtCHITV*) in leaves were up-regulated by *S. maltophilia*, a bacterium capable of secreting chitinases (Kępczyńska and Karczyński 2020). *P. fluorescens*, in which no chitinase activity was detected, up-regulates two genes, *MtCHITV* and *MtCHITIII*, like *S. maltophilia.* Salzer et al. (2000) showed a different expression of the *CHIT* genes in *M. truncatula* roots, depending on the contact with three biotic agents: a fungal pathogen (*Fusarium solani* sp. *phaseoli*), an arbuscular mycorrhiza (*Glomus intraradices*) and nodulation with *Rhizobium meliloti*. An enhanced expression of *MtCHITI, MtCHITII, MtCHITIII* and *MtCHITIV* was observed in roots in response to *Fusarium* strains; expression of *MtCHITI*, *MtCHITII* and Mt*CHITIV* was enhanced in symbiotic roots, *MtCHITIII* showing enhanced expression in mycorrhizal roots. Taken together, these findings show that biotic factors differ in their regulation of expression of the genes encoding chitinases representing two families; class I, II, IV chitinases belong to the glycosyl hydrolase family 19 (GH 19) (Santos et al. 2008), class III and V chitinases representing the GH18 family (Takenaka et al. 2009). Among the chitinases tested, only *CHITI* and *CHITV* have a chitin-binding domain (CBD) which is extremely important from the point of view of resistance to pathogens. The increased expression of other chitinases may be, however, useful also as they are involved at various stages of plant development (Grover 2012). The author referred that transcript expression of the chitinase genes is highly tissue- and organ-dependent. We demonstrated, too, that 4 out of the 5 chitinase genes studied in roots of the 4-week-old seedlings showed a high level of expression, compared to leaves; it was only expression of *MtCHITII* that was much higher in leaves than in roots. The high level of expression, in roots, of the *MtCHITI* and *MtCHITV* genes which show a chitin-binding domain may indicate that these chitinases are likely to serve not only these organs in protecting them against soil-borne fungal pathogens, but they may participate in the formation of signaling molecules that regulate the developmental processes (Kasprzewska 2003; van Loon et al. 2006). Moreover, regulation of the chitinase activity by phytohormones, including auxins, additionally suggests that the enzymes may be involved in development and growth processes (Umemoto et al. 2012). The role of chitinases in defense against fungal pathogens has been establised using, *inter alia*, genetic transformation; transgenic plants overexpressing chitinases and their enhanced resistance to pathogens including *Fusarium* species were listed by Grover (2012).

In this study, we also examined expression of the gene encoding β-1,3-glucanase (glucan-β-1,3-glucosidases; GLU), an enzyme which, particularly in combination with chitinases, participates in the lysis of fungal chitin-glucan fibers from cell walls. Earlier we demonstrated that the transgenic *Linum usitatissimum* overexpressed glucanase gene showed about threefold increase in resistance to the *Fusarium oxysporum* and *F.culmorum* (Wróbel-Kwiatkowska et al. 2004). In this study, we showed for the first time that the *MtGLU* and *MtCHITII* genes are very highly expressed in leaves of 4-week-old *M. truncatula*, which may suggest participation of both enzymes encoded by the genes mentioned in leaf development.

In the context of suitability of the bacteria tested to limit the development of *Fusarium* species in plants affected, the two bacteria had a potential to be used in developing preparations composed of a bacterial consortium, because both were capable of directly reducing the fungus growth (Fig. 1) by secreting β-1,3-glucanase (Fig. 2). Particularly *S. maltophilia* can increase expression of the *MtGLU* gene encoding β-1,3-glucanase (Fig. 3); thus, its influence may be indirect in controlling the fungi. Kim et al. (2015) suggested earlier that the volatiles of *Bacillus* sp. JS confer resistance against the soil-borne tobacco pathogens *Rhizotonia solani* and *Phytophthora nicotianae* through up-regulation of the *GLU* and acidic pathogenesis-related protein *PR3* genes encoding β-1,3-glucanase and chitinase, respectively.

PGPR inoculation of *Arabidopsis thaliana* has been shown to significantly enhance expression of genes encoding the biosynthetic enzymes of the phenylpropanoid pathway leading to the synthesis of phytoalexins or phenols, which have a defense function in plants, e.g., the reinforcement of plant cell walls, exhibit an antimicrobial activity and are involved in the synthesis of signaling compounds such as salicylic acid (Vogt 2010). In this study, we also checked expression of the 4 *PAL* genes in roots and leaves of 4-week-old *M. truncatula* seedlings. Earlier, Ren et al. (2019), who studied the genome of *M. truncatula* (Jemalong) A17 (the plant used also in our research), identified 6 *PAL* genes that encode PAL. Among the 4 *PAL* genes we selected 3 (*MtPAL1, MtPAL2, MtPAL4)* showed a higher expression level in 4-week-old *M. truncatula* seedling roots compared to leaves. Under the influence of the two bacteria tested, two genes: *MtPAL4* and *MtPAL5* were up-regulated in roots and leaves depending on time; expression of *MtPAL2* in leaves was enhanced by *S. maltophilia* only*.* In summary, the *MtPAL4* and *MtPAL5* genes, which significantly increased their expression under the influence of *P. fluorescens* Ms9N and *S. maltophilia* Ll4, may be markers of the interaction between *M. truncatula* and bacteria of the genera *Pseudomonas* and *Stenotrophomonas.* Abbasi et al. (2019) examined expression of only one *PAL1* gene and found the expression to be induced in tomato shoots after 12 days upon pre-treatment of the soil with two *Streptomyces* strains (IC10, Y28). In turn, Rahimi et al. (2020) showed that only in Fe-deficient lateral roots of the *Cydonia oblonga* seedlings treated with two siderophore-producing strains of PGPR, *P. fluorescens* and *Microccucuce yunnanensis* increase the *PAL1* gene expression.

### Changes in relative expression of several genes from MYB and WRKY families in *M. truncatula* roots and leaves after soil treatment

The TF-mediated gene expression regulatory networks play an important role in plant growth and development. The MYB proteins have been shown to be involved *inter alia* in the control of cell development and the cell cycle, flavonoid biosynthesis and in response to various abiotic and biotic stresses (Li et al. 2019a, b). Also WRKY TFs are modulated by abiotic and biotic factors (Li et al. 2004; Euglem and Somssich 2007; Jaśkiewicz et al. 2011; Mathys et al. 2012; Backer et al. 2019). Therefore, in this study we checked, for the first time, whether two different species of PGPR bacteria: *P. fluorescens* Ms9N and *S. maltophilia* Ll4 affect expression of the selected genes encoding TF proteins from MYB and WRKY families.

*MYB74* and *MYB102* belong to the R2R3-MYB class of myeloblastosis (MYB) genes which, in the *Medicago truncatula* genome, contains about 150 member genes (Li et al. 2019b). The search for the orthological protein of *Arabidopsis thaliana* MYB102 in *M. truncatula* was first undertaken by Wang and Li (2017). They showed both MYB74 and MYB102 of *M. truncatula* to form one clade and to be similar to *At*MYB102. Their analysis did not include MYB74, an MYB102 paralog. Our phylogenetic analysis showed both *Mt*MYB74 and *Mt*MYB102 to be more similar to their *A. thaliana* homologs than to each other (Fig. S1a). Both *MtMYB74* and *MtMYB102* were expressed in *M. truncatula* roots and leaves. The two different bacteria species elicit different responses in *M. truncatula* roots and leaves. The roots showed up-regulation of one of the two genes tested, *MtMYB102,* caused by one of the bacteria, *P. fluorescens.* However, both *P. fluorescens* Ms9N and *S*. *maltophilia* Ll4 up-regulated *MtMYB102* in leaves*,* but *MtMYB74* was up-regulated there only by *S*. *maltophilia.* Taken together, our data for *MtMYB74* and *MtMYB102* show that these genes can potentially play a key role in the interaction between *M. truncatula* and the beneficial bacteria. These results suggested that tested bacteria produced some signal which activated expression of the gene in leaves. Earlier, Stringlis et al. (2018) showed activation of *AtMYB72* following the *Pseudomonas simiae* WCS417 treatment of *Arabidopsis* seedlings roots to require auxin signaling. Our two bacteria do produce auxins (Kępczyńska and Karczyński 2020) caused up-regulation of *MtMYB102* in leaves, which confirms findings of Stringlis et al. (2018) with respect to *AtMYB72*. In turn, Ortiz-Garcia et al. (2022) showed the accumulation of indole-3-acetamide (IAM), an auxin precursor, in the *ami1* mutant reduced *A. thaliana* seedling growth and triggered abiotic stress responses. In addition, the authors referred to provided evidence that *MYB74* showed a stronger response to IAM, compared to *MYB102.* They suggested that MYB74 is a negative plant growth regulator, since conditional *MYB74* overexpression lines showed a considerable reduction of seedling growth. They also provided evidence for involvement of this TF in the regulation of a broad number of abiotic stress response-related processes. Earlier, Wang and Li (2017) identified 166 MYB TFs in *M. truncatula* according to the *A. thaliana* genome and, based on the phylogenetical analysis, divided them into 14 subgroups; most MYB TFs were involved in plant development, while *AtMYB102* (Subgroup 5) is associated with the defense function (de Vos et al. 2006; Zhu et al. 2018). In this study, the up-regulation of *MtMYB102* observed 24 h after soil inoculation with *P. fluorescens* Ms9N may be probably related to its involvement in *M. truncatula* root development. Previously, we showed that a 24-h contact with the suspension of this bacterial strain resulted in a several-fold increase in expression of *MtWOX5*, a known lateral root inducer, which is probably associated with initiation of a process leading to the formation of a very well-rooted system observed in *M. truncatula* seedlings (Kępczyńska and Karczyński 2020). The increased expression of this gene due to the presence of bacteria was accompanied by the cell cycle arrest in the S phase in the nuclei of both root tips and the lateral zone cells. In summary, our new data suggest that the two PGPR bacteria studied are capable of modulating expression of some *MYB* genes in *M. truncatula* roots and leaves, and a lot of work needs to be done to clarify the functions of these proteins in this fabacean molecular model. The role of other genes from the *MYB* family in root development and differentiation had been earlier reported by other workers. Shin et al. (2007) showed that expression of auxin-inducible genes was modulated by *AtMYB77* regulating lateral root formation, while Mu et al. (2009) found *AtMYB59* to regulate root development through controlling the cell cycle progression at the root tips.

The *WRKY* genes coding the WRKY family protein contribute, too, to the defense priming process induced by abiotic and biotic factors, known mainly in *Arabidopsis* (Eulgem and Somssich 2007; Jaśkiewicz et al. 2011; Cheng et al.^2012^; Ishihama and Yoshioka 2012; Chen et al. 2019). Our phylogenetic analysis of the 4 *WRKY* genes (*MtWRKY6, MtWRKY29, MtWRKY53* and *MtWRKY70*) we selected showed all the 4 M*. truncatula* WRKY proteins to be aligned with the orthological WRKY proteins found in *Arabidopsis*, and all showed the presence of the WRKY domain necessary for their proper functioning. Constitutive expression of the genes mentioned in *M. truncatula* took place in roots and leaves of 4 week–old seedlings and was organ-dependent. Up-regulation of the *MtWRKY6*, *MtWRKY29*, *MtWRKY53* and *MtWRKY70* genes in *M. truncatula* leaves after soil treatment with *S. maltophilia* and up-regulation of the *MtWRKY6, MtWRKY29* and *MtWRKY70* genes by *P. fluorescens* indicates that these genes may be systemic signals involved in the priming response. Up-regulation of the TFs *AtWRKY53* and *AtWRKY70* by the rhizobacterium *Bacillus cereus* AR156 in *Arabidopsis* leaves was reported to be associated with induction of systemic resistance against pathogens through activation of the salicylic acid (SA) signaling pathway (Nie et al. 2017; Wang et al. 2018). Up-regulation of *WRKY70* was also observed in leaves of tomato plants treated with SA or inoculated with the PGPR *Streptomyces* IC10 strain (Abbasi et al. 2019). All the findings referred to above show that, depending on the bacteria strain and organ, colonization of *M. truncatula* roots as a result of watering the soil with suspensions of *P. fluorescens* Ms9N and *S. maltophilia* Ll4 trigger a signal which increases expression of some selected genes (*MtMYB74*, *MtMYB102, MtWRKY6, MtWRKY29, MtWRKY53, MtWRKY70*) encoding proteins from the MYB and WRKY families.

Taken together, results of the molecular studies referred clearly suggest that two *M. truncatula* PGPR: *P. fluorescens* Ms9N and *S. maltophilia* Ll4 (Kępczyńska and Karczyński 2020), albeit not exerting the same effect on the expression level of the genes in roots and leaves may be taken into account as priming immune responses. This is because the bacteria participate in induction of the genes encoding glucanases, chitinases and PAL, proteins to be associated with systemic resistance. These bacteria upon soil treatment can also modified in the roots and leaves of *M. truncatula* expression of some genes encoding MYB and WRKY TFs widely distributed in higher plants and may serve as regulators of plant responses to different environmental factors. To date, such data for *M. truncatula*, a model plant for genetic research on legumes, are lacking.

Moreover, the bacteria studied directly limit the growth of soil fungal pathogens of the genus *Fusarium,* dangerous to various plant species, not only the fabacean. Additionally, the presented results show that both the direct and indirect action of *S. maltophilia* Ll4 is more effective than that of *P. fluorescens* Ms9N, which suggests that, to intensify the final effect, “biofungicides” should be developed based on at least two strains rather than on a single bacterial strain. Some of the genes encoding proteins known with the priming response that we use, may be taken into account when selecting bacteria with an antifungal potential.

#### *Author contribution statement*

EK conceived and designed the research, provided the funding, analyzed the results and wrote the manuscript. PK and AO conducted the experiments. AO performed genes identification and bioinformatic analysis. PK and AO carried out statistical analyses, prepared tables and figures and preliminary version of material and methods. All authors have read the manuscript.

## Supplementary Information

Below is the link to the electronic supplementary material.

Supplementary file1 (DOCX 86 KB)

Supplementary file2 (DOCX 23 KB)

## Data Availability

All data generated or analyzed during this study are included in this published article (and its supplementary information files).

## Abbreviations

CHIT: Chitinase
GLU: Glucanase
ISR: Induced systemic resistance
PAL: Phenylalanine ammonia lyase
PGPR: Plant growth-promoting rhizobacteria
SAR: Acquired systemic resistance
TF: Transcription factor
