# Supplementary material for: Two Medicago truncatula growth-promoting rhizobacteria capable of limiting in vitro growth of the Fusarium soil-borne pathogens modulate defense genes expression
Source: Planta. 2023 May 12;257(6):118. doi: 10.1007/s00425-023-04145-9 (PMC10181981; doi:10.1007/s00425-023-04145-9)
Supplement: Supplementary file 1 — Supplementary file1 (DOCX 86 KB) [file 425_2023_4145_MOESM1_ESM.docx]

**a**


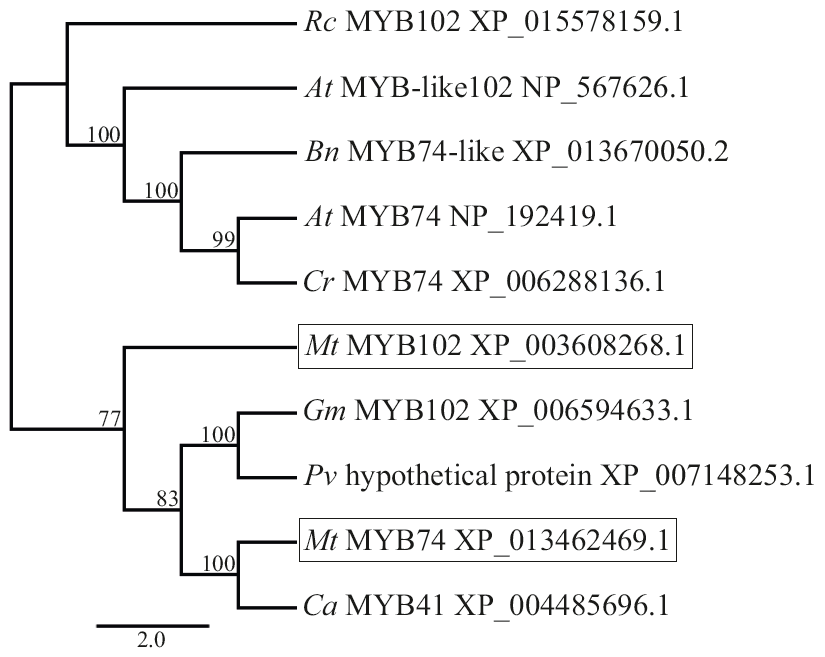


**b**


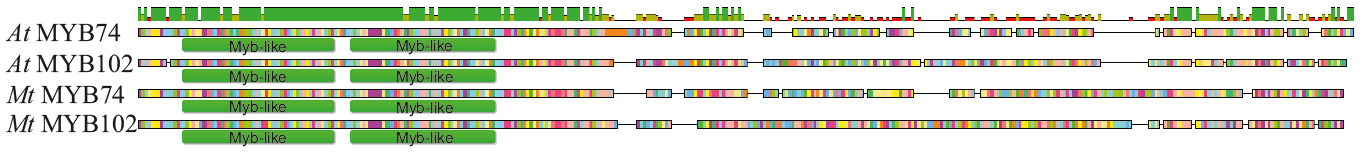


**Supplementary Figure S1.** Phylogenetic trees and domain organization based on deduced amino acid sequences of *Medicago truncatula* (*Mt*) and *Arabidopsis thaliana* (*At*) MYB74, MYB102 (a, b). Bn, *Brassica napus*; Ca, *Cicer arietinum*; Cr, *Capsella rubella*; Gm, *Glycine max*; Pv, *Phaseolus vulgaris*; Rc, *Ricinus communis*

**Two *Medicago truncatula* growth-promoting rhizobacteria capable of limiting *in vitro* growth of the *Fusarium* soil-borne pathogens modulate expression of some genes known as markers of defense pathways as well as some MYB and WRKY genes encoding transcriptional factors**

**Piotr Karczyński, Anna Orłowska, Ewa Kępczyńska*,**

Institute of Biology, University of Szczecin, Wąska 13, 71-413 Szczecin, Poland *ewa.kepczynska@usz.edu.pl
