# Supplementary material for: Two Medicago truncatula growth-promoting rhizobacteria capable of limiting in vitro growth of the Fusarium soil-borne pathogens modulate defense genes expression
Source: Planta. 2023 May 12;257(6):118. doi: 10.1007/s00425-023-04145-9 (PMC10181981; doi:10.1007/s00425-023-04145-9)
Supplement: Supplementary file 2 — Supplementary file2 (DOCX 23 KB) [file 425_2023_4145_MOESM2_ESM.docx]

**Supplementary Table S1.** Description and primer sequences of analyzed genes

| **Gene name short** | **Accession numer nucleotide/protein** | **Gene symbol/** **Locus tag** | **Primer sequence** |
| --- | --- | --- | --- |
| *CHIT I* | XM_024779517.2 XP_024635285.2 | LOC11414323 | F: GCGGAGCAATGCGGTAAAC  R: CCACAGTAGTCGCCGGTTGAT |
| *CHIT II* | XM_013590880.3  XP_013446334.1 | MTR_8g074335 | F: GGTGATGATGCCACCCGTAA  R: TATGGTCCGTCTGGTGCACTT |
| *CHIT III* | XM_003592107.4  XP_003592155.1 | MTR_1g099320 | F: ATTGAACTTGGTTCCACCGAAA R: TGAGGAGCAGCACCAAGATACA |
| *CHIT IV* | XM_003597500.4  XP_003597548.2 | MTR_2g099470 | F: CACTTGCCATAGCCTTTTTTATTATG  R: GTATTGGCTGCAACACACTCCTT |
| *CHIT V* | XM_013602691.3  XP_013458145.1 | MTR_4g116990 | F: CAAGCCGGAACTGTTGTTATCTG  R: TGCAACATCTTGACCAGGGTAGT |
| *GLU* | XM_003607271.4  XP_003607319.1 | MTR_4g076470 | F: TCCACCCAATGATGGTGCTT  R: TGTCTCAATTGCTCCGGGTC |
| *MYB74* | XM_013607015.3  XP_013462469.1 | MTR_2g011660 | F: AAAGTTGTCGTCTCCGTTGGA  R: CAAATGAAAACCGACCACGTT |
| *MYB102* | XM_003608220.3  XP_003608268.1 | MTR_4g091490 | F: AGGACCATGGACACAAGAGGA  R: GGGAGTAATCTCCAGTTGCC |
| *PAL1* | XM_003590423.3  XP_003590471.1 | MTR_1g064090 | F: AGCGCTTATGTTAAAGCCGC  R: GGACCAAGCCATTGAGGTGA |
| *PAL2* | XM_003591829.4  XP_003591877.1 | MTR_1g094780 | F: CACTTCAGAAGCCCAAACAAGATAG  R: TGTTGCGTATCGAATGACTTCAA |
| *PAL4* | XM_003618028.4  XP_003618076.1 | MTR_5g098720 | F: AGCAGGTCTGAGTTCTGGGTTCT  R: GAAGCCACACCAGATCCAACA |
| *PAL5* | XM_013594536.3  XP_013449990.2 | MTR_7g101395 | F: TCCTTCACAAATCAGGAAATTCACA  R: TGTTGTGTGGTGGTACCTAGA |
| *WRKY6* | XM_013607417.3  XP_013462871.1 | MTR_2g023930 | F: CGATCCTAATTTCACCGCTGTT  R: CATGGCCACCACCAATAATG |
| *WRKY29* | XM_013591658.3  XP_013447112.1 | MTR_8g098945 | F: AGCTACCCCTAAAGAGCCTGAAC  R: GCTGGAATTTGCAGATCTTGATG |
| *WRKY53* | XM_013591724.3  XP_013447178.1 | MTR_8g099350 | F: ACCGGAATCCCCAGTGTCTA  R: CATTTGGGCGCAACCTTTCT |
| *WRKY70* | XM_003623590.3  XP_003623638.1 | MTR_7g073430 | F: GTTGGCCGGAATTCCAAACT  R: ATCTGGTTGGTTGGCTACCT |

**Two *Medicago truncatula* growth-promoting rhizobacteria capable of limiting *in vitro* growth of the *Fusarium* soil-borne pathogens modulate expression of some genes known as markers of defense pathways as well as some MYB and WRKY genes encoding transcriptional factors**

**Piotr Karczyński, Anna Orłowska, Ewa Kępczyńska*,**

Institute of Biology, University of Szczecin, Wąska 13, 71-413 Szczecin, Poland *ewa.kepczynska@usz.edu.pl
